# Vespa Velutina Nigritorax: a new causative agent for anaphylaxis

**DOI:** 10.1186/2045-7022-5-S3-P43

**Published:** 2015-03-30

**Authors:** Ana Isabel Tabar, Sendy Chugo, Alejandro Joral, Maria Teresa Lizaso, Susana Lizarza, Maria Jose Alvarez-Puebla, Esozia Arroabarren, Catalina Vela, Manuel Lombardero

**Affiliations:** 1 Complejo Hospitalario de Navarra, Pamplona, Spain; 2 Hospital Universitario Donostia, San Sebastian, Spain; 3 Departamento de I+D. ALK-Abello, Madrid, Spain

## 

*Vespa Velutina* (Vespidae family, Genus Vespa) (*VV*) is a wasp whose origin is Asia. It was first found in Europe by the end of 2005 in southern France, even though its arrival probably took place in Bordeaux in a wood container one year before. Since then, they have spread throughout 32 departments in France, getting to Guipuzcoa by 2011 and are already present in Navarre, attacking beehives, causing damages and social concern.

## Objective

We describe the clinical and etiological study carried out in 8 patients who suffered reactions (7 Systemic and 1 Large Local reaction (LLR) after VV stings.)

## Material and methods

After a thorough history we performed:

• Skin tests with Apis mellifera, Vespula and Polistes dominulus spp commercial extracts

• Total IgE and baseline Tryptase determinations

• Specific IgE against full extract of Apis mellifera, Vespula spp, Polistes dominulus and Vespa crabro.

• Specific IgE against components using ImmunoCAP with Api m1, Pol d5, Ves Ves v1 and v5

• A sample of VV venom (Spring Mills, PA, USA) was obtained and a IgE - immunoblotting performed using the individual serum of the patients.

## Results

• 7 patients suffered systemic reactions grade IV and one a LLR.

• 5 patients had positive skin tests with other vespid venom extracts

• We detected specific IgE to complete extract from other vespids in 6 patients.

• All the patients but one with a LLR recognized a protein of 23 kDa in the venom of VV. This protein was specifically recognized by anti-Ves v5 antibodies, indicating that it was the VV antigen 5 (Ves ve5). The amino acid sequence of seven tryptic peptides to Ves ve5 showed 70-100% homology with Ves c5, 54-85% with Ves v5 and 40-81% with Pol d5 sequences.

## Conclusion

The detection of specific IgE against antigen 5 from other vespids (Ves c5, Ves v5 and Pol d5) suggest the presence of an antigen 5 in Vespa velutina that shows cross-reaction with the counterparts of the most frequents vespids. The question that remains to be answered is whether the available immunotherapy for these counterparts could be effective in these patients, since there is no possibility of treatment with the specific venom, not marketed.

